# A coronary artery aneurysm revealing a Behçet’s disease: a case report

**DOI:** 10.11604/pamj.2020.36.3.22373

**Published:** 2020-05-04

**Authors:** Sameh Ben Farhat, Mehdi Slim

**Affiliations:** 1 Department of Cardiology, Sahloul University Hospital, Sousse, Tunisia

**Keywords:** Behçet’s disease, coronary aneurysm, acute coronary syndrome

## Abstract

Behçet’s disease (BD) is a multisystemic chronic vasculitis characterized by its clinical polymorphism. It concerns mainly young men and generally appears between the third and the fourth decades. Cardiac involvement in Behçet’s disease is rare but represents a major prognostic factor. We report the case of a young man admitted in our department for the management of an acute coronary syndrome revealing a Behçet’s disease. Coronary angiography had shown a giant thrombosed aneurysm of the left coronary artery. Surgical treatment was successfully performed and the patient had a left anterior descending coronary artery bypass using the left internal mammary artery graft.

## Introduction

First described in 1937, Behçet’s disease (BD) is defined as a chronic multisystemic inflammatory condition affecting small, medium and large-caliber vessels [1, 2]. Its prevalence varies widely from one region to another and is particularly high in the Middle and Far East [3]. Genetic, environmental or autoimmune factors have been implicated in its etiopathogenesis, however, the exact etiology remains unclear [2]. The disease usually appears during the 3^rd^ or the 4^th^ decade with a peak frequency around the age of 30 [1]. Both men and women could be affected, but there is a slight male predominance [4]. Cardiac involvement in BD is uncommon, however, it is clearly associated with a poor prognosis [2]. We report the case of a young man admitted in our department for acute coronary syndrome secondary to a thrombosed aneurysm of the left anterior descending coronary artery and revealing an angiobehçet.

## Patient and observation

A 32-year-old man, with no cardiovascular risk factors, was admitted in our department for typical chest pain that had been exaggerated in the last week. In his past medical history, he had a left hip synovitis treated with corticosteroids and a recurrent oral aphthosis. Cardiovascular examination was unremarkable; however, bilateral ulcerated scrotal lesions were noted. Electrocardiography (EKG) showed a non-persistent ST segment elevation in the lateral leads. Laboratory data revealed elevated cardiac troponin and C-reactive protein at a concentration of 4.71 μg/L and 100 mg/L respectively while other blood tests were within the standard limits.

Transthoracic echocardiography (TTE) was normal. He received antithrombotic therapy followed by a coronary angiography that showed multiple aneurysms of the main diagonal branch and the left circumflex coronary artery. The largest one measured 8 mm and was located in the bifurcation of the left anterior descending coronary artery (LAD) with the first diagonal branch. BD was strongly suspected and the patient was transferred to medicine department where the diagnosis was confirmed. He received immunosuppressive therapy that induced total remission of his disease and complete resolution of angina.

**Figure 1:**
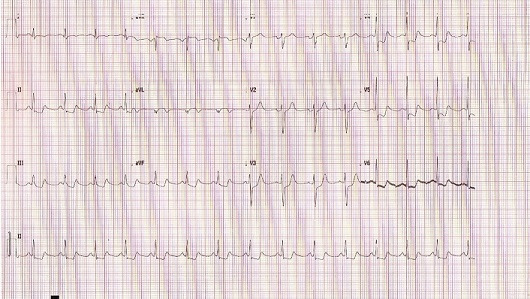
Electrocardiogram showing ST segment depression in inferior and apico-lateral leads

**Figure 2:**
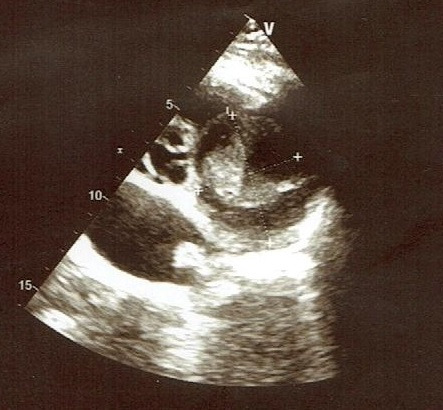
Transesophageal echocardiography, short axis view showing an aneurysmal mass contacting the pulmonary artery

**Figure 3:**
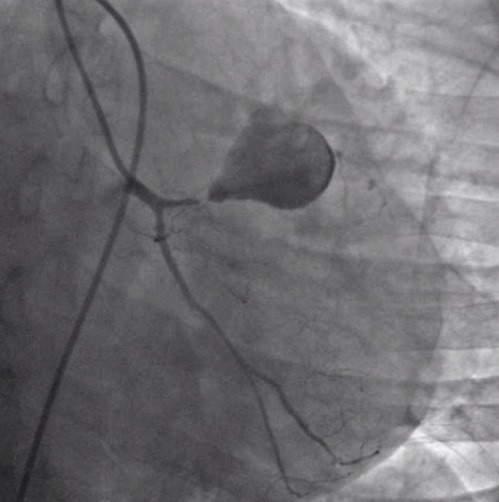
Coronary angiography revealing a thrombosed aneurysm of the left coronary artery

**Figure 4:**
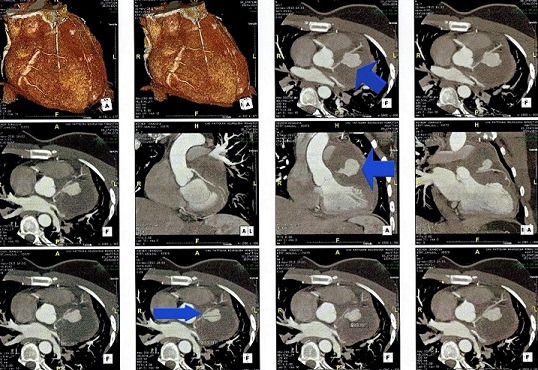
CT angiography identifying a saccular thrombosed aneurysm of the proximal segment of the LAD

A year later, he was readmitted for unstable angina. His physical examination was strictly normal. EKG showed regular sinus rhythm with ST segment depression in inferior and apicolateral leads (Figure 1). Blood tets revealed biological inflammatory syndrome but no elevation in the level of the myocardial markers of necrosis. Transesophageal echocardiography (TEE) revealed preserved biventricular function. Though, there was a huge aneurysmal mass measuring approximately 58*47 mm, arising from the left coronary artery and distorting the lateral wall of the left ventricle (LV) and the pulmonary artery (PA) without creating an authentic right outflow tract obstruction (Figure 2). We performed coronary angiography that showed a severe stenotic lesion of the proximal segment of the LAD localized opposite to a huge aneurysm of about 3 cm in size inducing a coronary steal phenomenon with a delayed opacification of the distality of the vessel (Figure 3). On computed tomography (CT) angiography, there was a saccular thrombosed aneurysm of the proximal segment of the LAD measuring 3cm with a neck portion of 4.3 mm (Figure 4). Two other small aneurysms of the second diagonal branch of 3 and 4 mm of diameter respectively were also noted.

**Figure 5:**
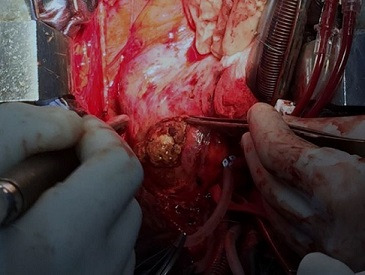
Peroperative view of the thrombus filling the aneurysmal sac

Surgery was performed after inflammation control. Peroperative exploration identified the aneurysmal sac filled with a big thrombus and contacting the lateral wall of the LV and the PA (Figure 5). Surgeons proceeded to open and resect the aneurysm then sutured the neck portion. Afterward they performed a bypass of the LAD using the left internal mammary artery graft. The postoperative period went without incident and the patient was successfully discharged.

## Discussion

Cardiac involvement is rare but severe during BD. Its prevalence varies from 0.36 to 19.5 per 100,000 inhabitants depending on the clinical series [1, 5]. All three layers of the cardiac wall can be affected. Thus, cardiac manifestations may include, pericarditis (20 to 40%), myocarditis (20%), endocarditis, endomyocardial fibrosis or myocardial infarction [6]. The coronary arteries are rarely involved (prevalence of 0.5%) by stenotic lesions, arteritis or especially aneurysms formation [4].

Physiologically, vascular involvement could be explained by the deposition of immune complexes in the vascular wall leading to a subsequent complement-activated leukocyte infiltration [4]. The inflammatory infiltrate made of plasma cells and lymphocytes affects preferably the media, the adventitia and the vasa-vasorum causing an abnormal weakening and thinning of the blood vessels wall with the formation of aneurysms and pseudoaneurysms which constitutes an authentic arterial aphtosis [1].

Behçet disorder is rarely inaugurated by coronary artery disease. In fact, most cases reported in the literature were already diagnosed and treated for this condition [2]. Typical clinical picture is represented by acute coronary syndrome and the first case was reported by Schiff *et al.* in 1982 [7]. Several mechanisms have been described in the literature: coronary occlusions secondary to vasculitis, the formation of thrombus in situ, compression of the vessels by an aneurysm or dilated valsalva sinus or less frequently an impairment of the microvascular function [2, 8]. The modalities for the management of coronary artery disease during BD are based on the decision of a multidisciplinary team including the internist, the cardiologist and the cardiovascular surgeon. In general, in addition to standard anti-ischematic therapy, the combination of corticosteroids with immunosuppressive drugs is highly recommended [9].

Complete remission can be achieved by medical treatment alone and some aneurysms may even completely regress. Nevertheless, surgical or endovascular approaches are deemed necessary in most circumstances. Surgery is recommended for giant aneurysms (size greater than 2 cm), with fast progression and high risk of rupture [3]. In other cases, surgical or interventional management can be discussed [4, 10, 11]. These two techniques may, however, carry more risk of disease progression which is inherent to their pathergy-like effects [12]. Several measures can reduce this risk, such as optimal control of inflammation in the preoperative period, careful examination of arterial or venous grafts before their implantation, and avoiding excessive manipulation of the aorta [2, 12].

## Conclusion

Coronary involvement during BD is rare but serious. Angiographically, the most commonly found coronary lesions are aneurysms for which treatment could be surgical or endovascular. Due to the unpredictable evolution of this condition and insufficient data concerning the medium and long-term results of both surgical and interventional techniques, long-term follow-up is required.

## Competing interests

The authors declare no competing interests.
